# Garlic's ability to prevent *in vitro *Cu^2+^-induced lipoprotein oxidation in human serum is preserved in heated garlic: effect unrelated to Cu^2+^-chelation

**DOI:** 10.1186/1475-2891-3-10

**Published:** 2004-09-01

**Authors:** José Pedraza-Chaverrí, Mariana Gil-Ortiz, Gabriela Albarrán, Laura Barbachano-Esparza, Marta Menjívar, Omar N Medina-Campos

**Affiliations:** 1 Departamento de Biología, Facultad de Química, Edificio B, Segundo Piso, Laboratorio 209, Universidad Nacional Autónoma de México (UNAM), Ciudad Universitaria, 04510, México, D.F., México

## Abstract

**Background:**

It has been shown that several extracts and compounds derived from garlic are able to inhibit Cu^2+^-induced low density lipoprotein oxidation. In this work we explored if the ability of aqueous garlic extract to prevent *in vitro *Cu^2+^-induced lipoprotein oxidation in human serum is affected by heating (a) aqueous garlic extracts or (b) garlic cloves. In the first case, aqueous extract of raw garlic and garlic powder were studied. In the second case, aqueous extract of boiled garlic cloves, microwave-treated garlic cloves, and pickled garlic were studied. It was also studied if the above mentioned preparations were able to chelate Cu^2+^.

**Methods:**

Cu^2+^-induced lipoprotein oxidation in human serum was followed by the formation of conjugated dienes at 234 nm and 37°C by 240 min in a phosphate buffer 20 mM, pH 7.4. Blood serum and CuSO_4 _were added to a final concentration of 0.67% and 0.0125 mM, respectively. The lag time and the area under the curve from the oxidation curves were obtained. The Cu^2+^-chelating properties of garlic extracts were assessed using an approach based upon restoring the activity of xanthine oxidase inhibited in the presence of 0.050 mM Cu^2+^. The activity of xanthine oxidase was assessed by monitoring the production of superoxide anion at 560 nm and the formation of uric acid at 295 nm. Data were compared by parametric or non-parametric analysis of variance followed by a post hoc test.

**Results:**

Extracts from garlic powder and raw garlic inhibited in a dose-dependent way Cu^2+^-induced lipoprotein oxidation. The heating of garlic extracts or garlic cloves was unable to alter significantly the increase in lag time and the decrease in the area under the curve observed with the unheated garlic extracts or raw garlic. In addition, it was found that the garlic extracts were unable to chelate Cu^2+^.

**Conclusions:**

(a) the heating of aqueous extracts of raw garlic or garlic powder or the heating of garlic cloves by boiling, microwave or pickling do not affect garlic's ability to inhibit Cu^2+^-induced lipoprotein oxidation in human serum, and (b) this ability is not secondary to Cu^2+^-chelation.

## Background

Garlic (*Allium sativum*) has been cultivated since ancient times and used as a spice and condiment for many centuries [1]. During the past years, there has been a growing awareness of the potential medicinal uses of garlic [2-5]. The antioxidant properties of garlic are well documented [6-8]. In particular, aqueous garlic extract [9] and aged garlic extract [10-12] are able to prevent Cu^2+^-induced low density lipoprotein (LDL) oxidation. In addition, Ide *et al*. [10] and Ho *et al*. [13] have shown that some garlic compounds such as S-allylcysteine, N-acetyl-S-allylcysteine, S-allylmercaptocysteine, alliin, and allixin, are also able to prevent Cu^2+^-induced LDL oxidation. Ou *et al*. [14] showed that the garlic compounds S-ethylcysteine, N-acetylcysteine, diallyl sulfide, and diallyl disulfide inhibit amphotericin- and Cu^2+^-induced LDL oxidation. Huang *et al*. [15] showed that diallyl sulfide, diallyl disulfide, S-allylcysteine, S-ethylcysteine, S-methylcysteine, and S-propylcysteine are able to prevent glucose-induced lipid oxidation in isolated LDL. The protective effect of aged garlic extract [10,11] and S-ethylcysteine, N-acetylcysteine, diallyl sulfide, and diallyl disulfide [14] on Cu^2+^-induced LDL oxidation may be explained, at least in part, for their ability to chelate Cu^2+^. Interestingly, the diethyl ether extract of aged garlic extract, which also inhibits Cu^2+^-induced LDL oxidation, is unable to chelate Cu^2+ ^[11] indicating that its ability to prevent LDL oxidation is unrelated to Cu^2+^-chelation.

On the other hand, 95% of the sulfur in intact garlic cloves is found in two classes of compounds in similar abundance: the S-alkylcysteine sulfoxides and the γ-glutamyl-S-alkylcysteines [16]. The most abundant sulfur compound in garlic is alliin (S-allylcysteine sulfoxide), which is present at 10 mg/g fresh garlic or 30 mg/g dry weight [16]. When garlic cloves are cut, crushed, or chopped (or when the powder of dried cloves becomes wet in a non-acid solution), the cysteine sulfoxides, which are odorless, are very rapidly converted to a new class of compounds, the thiosulfinates which are responsible for the odor of freshly chopped garlic. The formation of thiosulfinates takes place when the cysteine sulfoxides, which are located only in the clove mesophyll storage cells, come in contact with the enzyme allinase or alliin lyase (E.C. 4.4.1.4), which is located only in the vascular bundle sheath cells. Allinase is active at pH 4–5.8, but is immediately inhibited at acidic pH values below 3.5 or by cooking. Furthermore, microwave heating destroys allinase activity in 1 min [17]. Due to the abundance of alliin, the main thiosulfinate formed upon crushing garlic is allicin [16]. The half-life of allicin at room temperature is 2–16 hours; however, in crushed garlic (or in garlic juice) it is 2.4 days [16].

Several studies have been performed to test the effect of heating on several garlic properties. It has been shown that the boiling of garlic cloves by 15 min impairs significantly its ability to inhibit cyclooxygenase activity [18] and thromboxane B_2 _synthesis [19]. In addition, heating of garlic cloves by 60 seconds in microwave reduces its anticancer properties [17]. Interestingly when microwave heating was applied 10 minutes after garlic crushing the anticancer properties were preserved indicating that allinase activation is necessary to generate anticancer compounds which are heat stable [17]. In a similar way, the hydroxyl scavenging properties of garlic were essentially preserved when garlic extracts were heated at 100°C by 20, 40 or 60 min [20]. In contrast, heating of garlic extracts by 10 min at 100°C reduced the bactericidal activity against *Helicobacter pylori *[21] and the ability to inhibit platelet aggregation [22]. However, to our knowledge, there are no studies exploring if the heating of garlic cloves or aqueous extract of raw garlic or garlic powder are able to inhibit Cu^2+^-induced lipoprotein oxidation in human serum. In the present paper we studied if the ability of aqueous garlic extracts to inhibit *in vitro *Cu^2+^-induced lipoprotein oxidation in human serum is altered in the following aqueous preparations: (a) heated extract of garlic powder, (b) heated extract of raw garlic, (c) extract of boiled garlic cloves, (d) extract of microwave-treated garlic cloves, and (e) extract of pickled garlic. In addition it was studied if the above mentioned preparations are able to chelate Cu^2+^.

It was found that (a) the heating of garlic extracts or garlic cloves had no influence on the ability of garlic extracts to prevent *in vitro *Cu^2+^-induced lipoprotein oxidation in human serum, and (b) this protective effect was unrelated to Cu^2+^-chelation.

## Methods

### Materials and reagents

Bulbs of garlic were from a local market. Garlic powder was from McCormick (Mexico City, Mexico). Copper sulfate, Na_2_EDTA, Na_2_CO_3_, KH_2_PO_4 _, and Na_2_HPO_4 _were from JT Baker (Mexico City, Mexico). Copper sulfate was dissolved in distilled water. Xanthine oxidase, xanthine, and nitroblue tetrazolium (NBT) were from Sigma Chemical Co. (St. Louis, MO., USA).

### Preparation of aqueous extracts of garlic

#### Extract of garlic powder (GP)

Garlic powder was weighted (0.6 g), dissolved, and stirred with 6 mL of distilled water for 20 min. This solution was centrifuged at 20,124 × g for 5 min at 4°C. The supernatant was recovered and used at the final concentration of 0.05, 0.075, 0.10, and 0.25 mg/mL.

#### Heated extract of garlic powder (HGP)

The procedure was similar to the previous one except that the mixture was boiled for 20 min before the centrifugation. The supernatant was recovered and used at the final concentration of 0.05 and 0.10 mg/mL.

#### Extract of raw garlic (RG)

Garlic cloves were peeled off, weighted, chopped, and homogenized with distilled water in a Polytron (Model PT2000, Brinkmann, Switzerland). This homogenate was centrifuged at 1,277 × g for 10 min and the supernatant was centrifuged at 20,124 × g for 5 min at 4°C. The supernatant was recovered and used at the final concentration of 0.125, 0.25, 0.5, and 0.75 mg/mL.

#### Heated extract of raw garlic (HRG)

The procedure was similar to the previous one except that the homogenate was boiled for 20 min before the second centrifugation step. The amount of water evaporated was replaced at the end of the heating. The supernatant was recovered and used at the final concentration of 0.25 and 0.5 mg/mL.

#### Extract of boiled garlic (BG)

Unpeeled garlic cloves were boiled in water for 10 min. After this time, garlic cloves were peeled off and the aqueous extract was prepared as described before (extract of raw garlic). The supernatant was recovered and used at the final concentration of 0.25 and 0.5 mg/mL.

#### Extract of garlic cloves submitted to microwave heating (MG)

Unpeeled garlic cloves were submitted to microwave heating for 1 min (1100 watts). After this time, garlic cloves were peeled off and the aqueous extract was prepared as described before (extract of raw garlic). When allinase is inactivated by heating, the cascade of thiosulfinate formation is blocked from alliin, and allicin and its derivates can not be formed. It has been shown that as little as 60 seconds of microwave heating can totally destroy allinase enzyme activity whereas microwave heating for 30 seconds inhibits 90% of allinase activity compared with unheated garlic [17]. The supernatant was recovered and used at the final concentration of 0.25 and 0.5 mg/mL.

#### Preparation of pickled garlic (PG)

Garlic cloves were peeled off carefully to avoid allinase activation and put in an aqueous solution of vinegar (1:1, v/v) and then heated to the boiling point for 30 min. Garlic cloves were put into jars with same solution and then pasteurized for 5 min at 72°C. The jars were closed immediately and stored at 4°C. The experiments with pickled garlic were performed five weeks after. The aqueous extract was prepared as described before (extract of raw garlic). The supernatant was recovered and used at the final concentration of 0.25 and 0.5 mg/mL.

#### Blood collection

Blood samples were obtained from 2 male and 3 female healthy volunteers aged 24 to 46 years. The experimental protocol is in compliance with the Helsinki Declaration and written informed consent was obtained from all subjects. A fasting blood sample was drawn from the antecubital fossa vein into glass tubes, allowed to clot, and then centrifuged at 2,000 × g for 10 min. The serum removed was aliquoted and frozen at -80°C until assayed. The concentration of glucose, cholesterol and triglycerides in serum was measured by an autoanalyzer (Hitachi 917 Automatic Analyzer, Boheringer Mannheim Corporation, Indianapolis, IN, USA).

#### Cu^2+^-induced lipoprotein oxidation in human serum

A modification of the serum oxidation method described by Regnstrom *et al*. [23] was used. This method provides an indication of conjugated dienes formation in lipoprotein fatty acids present in serum exposed to Cu^2+^, assessed by measuring changes in absorbance at 234 nm. The formation of conjugated dienes in lipoprotein deficient serum exposed to Cu^2+ ^is absent, indicating that diene formation in lipoprotein fatty acids is primarily responsible for the increase in absorbance [23]. The oxidation curves have three phases: lag, propagation, and decomposition. This method has been used previously by others [24-28]. Serum was diluted to a final concentration of 0.67% in 20 mM phosphate buffer, pH 7.4 and saturated with O_2_. Oxidation was initiated by the addition of CuSO_4 _to a final concentration of 0.0125 mM. The formation of conjugated dienes was followed by monitoring the change in absorbance at 234 nm at 37°C on a Beckman DU-64 spectrophotometer equipped with a six position automatic sample changer (Fullerton, CA, USA). Absorbance readings were taken every 10 min over 240 min. The aqueous garlic extracts were added at the indicated concentrations (ranging from 0.05 to 0.75 mg/mL). Control tubes consisted of identical assays conditions but without the garlic extract. Since the transition from lag phase to propagation phase was continuous, lag time was defined as the intercept of the tangents of the propagation and lag phases and expressed in minutes.

#### Determination of Cu^2+ ^chelation of garlic extracts

The Cu^2+ ^chelating properties of aqueous garlic extracts were assessed using an approach based upon restoring the activity of xanthine oxidase described previously [11]. This enzyme is inhibited in the presence of 0.050 mM CuSO_4 _[29]. The activity of xanthine oxidase can be assessed by monitoring either the production of superoxide anion or the formation of uric acid. Xanthine oxidase activity would be restored if garlic extracts were able to chelate Cu^2+^.

Xanthine oxidase activity was measured by NBT reduction and uric acid production. The following concentrated solutions were prepared in distilled water: xanthine oxidase 168 U/L, xanthine 0.30 mM, NBT 0.15 mM, and Na_2_CO_3 _0.4 M. Superoxide anions were generated in a reaction volume of 1 mL containing in a final concentration: xanthine 0.087 mM, Na_2_CO_3 _15 mM, NBT 0.022 mM, and 50 mM phosphate buffer pH 7.4 or garlic extract in a volume of 0.1 mL for control or experimental tube, respectively. In additional tubes with or without garlic extract, CuSO_4 _was added in a final concentration of 0.050 mM. The reaction was initiated by the addition of 0.025 U of xanthine oxidase, and superoxide anion production was monitored at 560 nm. In the same experiment, xanthine oxidase activity was measured by following the uric acid production at 295 nm [30]. Absorbance at 560 and 295 nm was obtained every minute for 3 minutes and the results were expressed as change of absorbance/min. The garlic extracts were added at the higher concentration used in the experiments for Cu^2+^-induced lipoprotein oxidation along with (+CuSO_4_) or without (-CuSO_4_) 0.050 mM CuSO_4_. Control tubes contained all the reagents but without garlic extracts and they considered as 100% production of uric acid and superoxide anion. In a separate tube with 0.050 mM CuSO_4_, 0.060 mM EDTA was added as an additional control in which was expected the restoration of xanthine oxidase since EDTA is a metal-chelating agent.

### Statistics

Data are expressed as mean ± SEM of five determinations using different serum samples. The variables used to describe the difference between the oxidation curves were lag time, area under the oxidation curve (AUC), and slope of the propagation phase. These parameters were obtained using the GraphPad Prism software v. 3.02 (San Diego, CA, USA). All parameters were compared using either one way analyses of variance followed by Bonferroni's t test or Kruskall-Wallis analysis of variance followed by Dunn's test. p < 0.05 was considered significant.

## Results

### Glucose, cholesterol, and triglycerides in human serum

The concentration, in mmol/L, of glucose, cholesterol, and triglycerides in serum, of the subjects involved in this study was 4.56 ± 0.15, 4.24 ± 0.42, and 1.05 ± 0.11, respectively. These data show that the human beings from whom the blood serum samples were obtained for this study had no alterations in the circulating levels of glucose, cholesterol, and triglycerides.

### Effect of garlic extracts on Cu^2+^-induced lipoprotein oxidation in human serum

**Figure 1:**
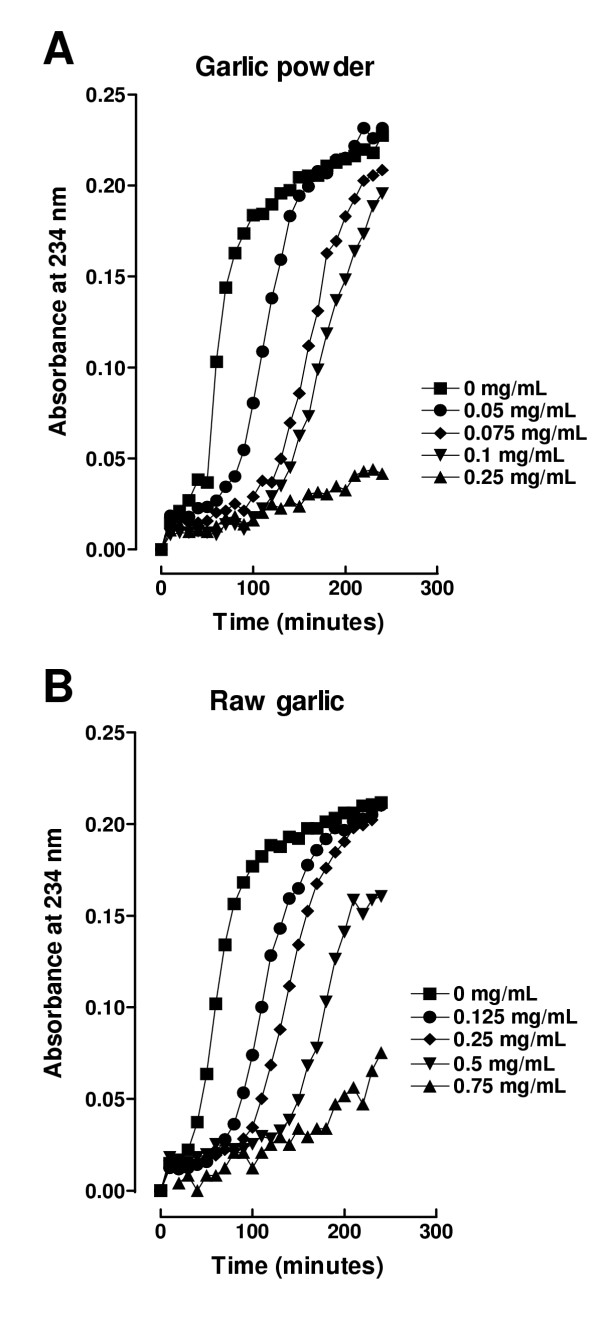
Representative curves showing the effect of aqueous garlic extracts on Cu^2+^-induced lipoprotein oxidation in human serum. Panel A shows the effect of garlic powder extract and panel B shows the effect of raw garlic extract. Cu^2+^-induced oxidation was followed at 234 nm. Readings were taken every 10 min. Oxidation was started by the addition of CuSO_4 _at a final concentration of 0.0125 mM in 20 mM phosphate buffer, pH 7.4 saturated with O_2 _and the readings were followed by 240 min at 37°C.

**Figure 2:**
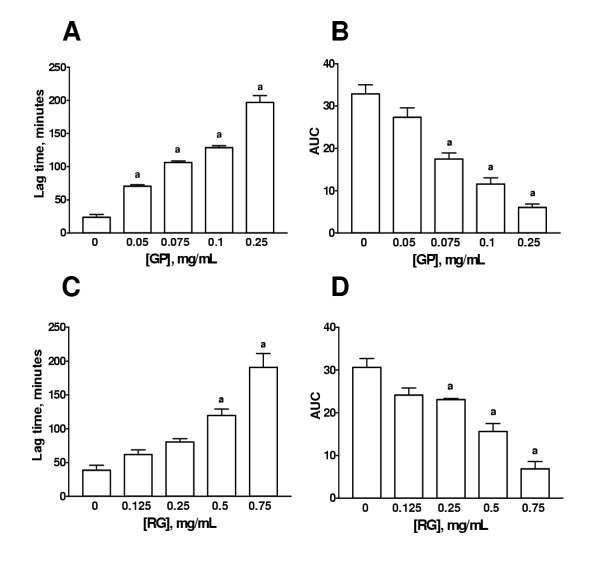
Dose-dependent effect of aqueous garlic extracts on lag time and AUC. Panels A and B shows the effect of garlic powder extract and panels C and D shows the effect of raw garlic extract. Lag time is shown in panels A and C and AUC is shown on panels B and D. ^a^p < 0.01 (panel A) and p < 0.001 (panels B, C, and D) vs. 0 mg/mL. Data are mean ± SEM of five determinations using independent samples.

Unheated garlic extracts from raw garlic or from garlic powder inhibited lipoprotein oxidation in a dose-dependent way. Figure 1 shows a representative graph obtained from a single subject. Panel A shows the effect of increasing concentrations of garlic powder extract (0.05 to 0.25 mg/mL) and panel B shows the effect of increasing concentrations of raw garlic extract (0.125 to 0.75 mg/mL) on Cu^2+^-induced lipoprotein oxidation in human serum. The increasing concentrations of garlic extracts displaced the curve to the right, compared to the control curve obtained without garlic extract, indicating that the inhibition of Cu^2+^-induced lipoprotein oxidation is dose-dependent. Lag time, AUC, and slope were obtained from these oxidation curves. Figure 2 shows the lag time (panels A and C) and AUC (panels B and D) and Table 1 shows the slopes of the propagation phase from the five subjects studied. Panels A and B show the effect of garlic powder extract and panels C and D show the effect of raw garlic extract. It can be seen that garlic extracts dose-dependently increased lag time and decreased AUC and slopes. Based on these curves the concentrations of all the extracts studied were chosen: 0.25 and 0.5 mg/mL for HRG, BG, MG, and PG; and 0.05 and 0.1 mg/mL for GP and HGP.

**Table 1:** Effect of extract of garlic powder (GP) and raw garlic (RG) on the slopes of oxidation curves.

GP, [mg/mL]	0	0.05	0.075	0.1	0.25
slope	0.0025 ± 0.0002	0.0017 ± 0.0003	0.0014 ± 0.0002	0.0009 ± 0.0003	0.0003 ± 0.00003^a^

RG, [mg/mL]	0	0.125	0.25	0.5	0.75

slope	0.0027 ± 0.0002	0.0018 ± 0.0002	0.0022 ± 0.0002	0.0017 ± 0.0002^b^	0.0007 ± 0.0003^a^

Data are mean ± SEM of five determinations using independent samples. ^a^p < 0.001, ^b^p < 0.05 vs. 0 mg/mL.

### Effect of different treatments of garlic on Cu^2+^-induced lipoprotein oxidation in human serum

**Figure 3:**
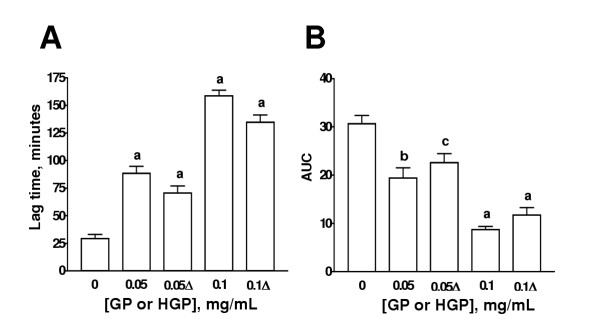
Effect of heated extract of garlic powder (HGP) on lag time and AUC. Aqueous extract of garlic powder was heated at 100°C by 10 min. Aqueous extracts of unheated (GP) and HGP were added to the system at two concentrations (0.25 and 0.5 mg/mL). Cu^2+^-induced oxidation was followed at 234 nm. Readings were taken every 10 min. Oxidation was started by the addition of Cu^2+ ^at a final concentration of 0.0125 mM in 20 mM phosphate buffer, pH 7.4 saturated with O_2 _and the readings were followed by 240 min at 37°C. ^a^p < 0.001, ^b^p < 0.01, and ^c^p < 0.05 vs. 0 mg/mL. Δ= HGP. Data are mean ± SEM of five determinations using independent samples.

**Figure 4:**
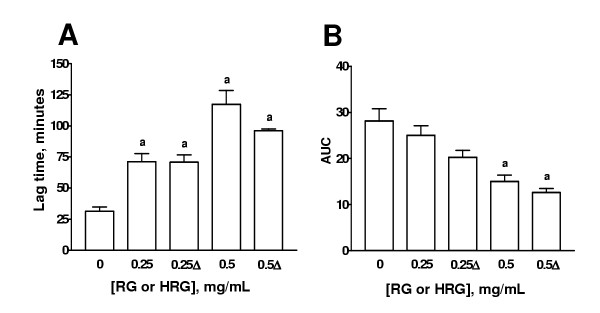
Effect of heated extract of raw garlic (HRG) on lag time and AUC. Aqueous extract of raw garlic was heated at 100°C by 10 min. Aqueous extracts of unheated (RG) or HRG  were added to the system at two concentrations (0.25 and 0.5 mg/mL). Cu^2+^-induced oxidation was followed at 234 nm. Readings were taken every 10 min. Oxidation was started by the addition of Cu^2+ ^at a final concentration of 0.0125 mM in 20 mM phosphate buffer, pH 7.4 saturated with O_2 _and the readings were followed by 240 min at 37°C. ^a^p < 0.05 vs. 0 mg/mL. Δ= HRG. Data are mean ± SEM of five determinations using independent samples.

**Figure 5:**
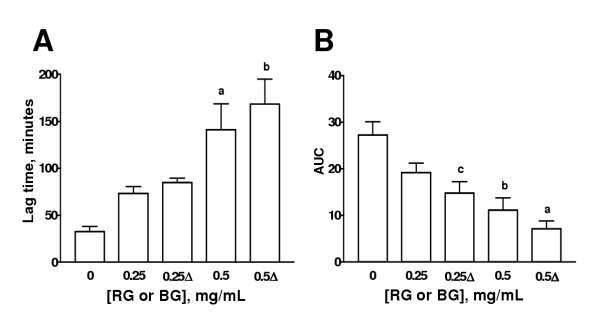
Effect of aqueous extract of boiled garlic (BG) cloves on lag time and AUC. Comparison was made with aqueous extract of raw garlic (RG). Aqueous extracts of BG and RG were added to the system at two concentrations (0.25 and 0.5 mg/mL). Cu^2+^-induced oxidation was followed at 234 nm. Readings were taken every 10 min. Oxidation was started by the addition of Cu^2+ ^at a final concentration of 0.0125 mM in 20 mM phosphate buffer, pH 7.4 saturated with O_2 _and the readings were followed by 240 min at 37°C. ^a^p < 0.001, ^b^p < 0.01, and ^c^p < 0.05 vs. 0 mg/mL. Δ= BG cloves. Data are mean ± SEM of five determinations using independent samples.

**Figure 6:**
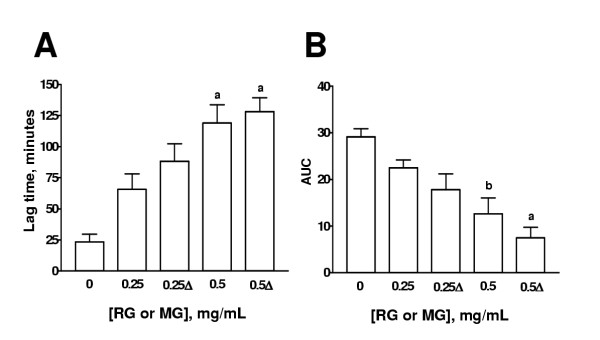
Effect of aqueous extract of garlic cloves submitted to microwave heating (MG) on lag time and AUC. Comparison was made with aqueous extract of raw garlic (RG). Aqueous extracts of microwave-treated garlic cloves (MG) and RG were added to the system at two concentrations (0.25 and 0.5 mg/mL). Cu^2+^-induced oxidation was followed at 234 nm. Readings were taken every 10 min. Oxidation was started by the addition of Cu^2+ ^at a final concentration of 0.0125 mM in 20 mM phosphate buffer, pH 7.4 saturated with O_2 _and the readings were followed by 240 min at 37°C. ^a^p < 0.001 and ^b^p < 0.01 vs. 0 mg/mL. Δ= MG. Data are mean ± SEM of five determinations using independent samples.

**Figure 7:**
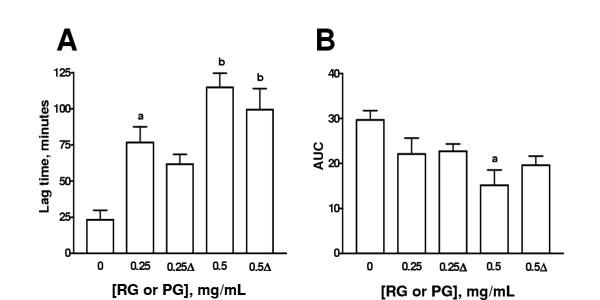
Effect of aqueous extract of pickled garlic (PG) on lag time and AUC. Comparison was made with aqueous extract of raw garlic (RG). Aqueous extracts of PG and RG were added to the system at two concentrations (0.25 and 0.5 mg/mL). Cu^2+^-induced oxidation was followed at 234 nm. Readings were taken every 10 min. Oxidation was started by the addition of Cu^2+ ^at a final concentration of 0.0125 mM in 20 mM phosphate buffer, pH 7.4 saturated with O_2 _and the readings were followed by 240 min at 37°C. ^a^p < 0.05, ^b^p < 0.001 (panel A), and ^a^p < 0.01 (panel B) vs. 0 mg/mL. Δ= PG. Data are mean ± SEM of five determinations using independent samples.

The effect of HGP, HRG, BG, MG, and PG on the lag time and AUC is shown on Figs. 3,4,5,6,7 respectively. The effects of these extracts on the slopes of oxidation curves are shown in Tables 2 and 3. GP and HGP were studied at 0.05 and 0.1 mg/mL and HRG, BG, MG, and PG were studied at 0.25 and 0.5 mg/mL. The data of HGP and HRG were compared with those of unheated extracts, GP and RG, respectively (Figs. 3 and 4 and Tables 2 and 3). The data of BG, MG, and PG were compared with those of RG (Figs. 5,6,7 and Table 3). It can be seen that the extracts increased lag time and decreased AUC at both concentrations studied indicating that they inhibit Cu^2+^-induced lipoprotein oxidation. The decrease in the slope was significant only at the higher concentration for GP and HGP (Table 2) and for RG and HRG, BG, and MG (Table 3). The decrease in the slope in PG was not significant (Table 3). Interestingly, the treatments (heating of extracts of garlic powder or raw garlic or heating garlic cloves by boiling, microwave or pickling) had no significative effect on lag time, AUC, and slope. All the comparisons between unheated and heated extracts were not different. Our data show that the antioxidant ability of garlic on Cu^2+^-induced lipoprotein oxidation in human serum is not significantly affected by the above mentioned treatments.

**Table 2:** Effect of extract of garlic powder (GP) and heated garlic powder (HGP) on the slopes of oxidation curves.

Extract [mg/mL]	0	0.05	0.05 Δ	0.1	0.1 Δ
GP or HGP	0.0024 ± 0.0002	0.0017 ± 0.0002	0.0018 ± 0.0002	0.0013 ± 0.0001^a^	0.0013 ± 0.0001^a^

Data are mean ± SEM of five determinations using independent samples. ^a^p < 0.01 vs. 0 mg/mL. Δ = HGP.

**Table 3:** Effect of different extracts of garlic on the slopes of oxidation curves.

Extract, [mg/mL]	0	0.25	0.25 Δ	0.5	0.5 Δ
RG or HRG	0.0023 ± 0.0003	0.0020 ± 0.0001	0.0014 ± 0.0001^a^	0.0013 ± 0.002^b^	0.0011 ± 0.0002^b^
RG or BG	0.0021 ± 0.0001	0.0016 ± 0.0002	0.0008 ± 0.0002^c^	0.0007 ± 0.0004^c^	0.0005 ± 0.0001^c^
RG or MG	0.0024 ± 0.0002	0.0018 ± 0.0002	0.0015 ± 0.0002	0.0011 ± 0.0003^b^	0.0005 ± 0.0002^c^
RG or PG	0.0026 ± 0.0002	0.0023 ± 0.0004	0.0021 ± 0.0004	0.0016 ± 0.0003	0.0019 ± 0.0003

Data are mean ± SEM of five determinations using independent samples. ^a^p < 0.05, ^b^p < 0.01, ^c^p < 0.001, vs. 0 mg/mL. Δ = HRG, BG, MG or PG.

### Cu^2+^-chelation studies

**Figure 8:**
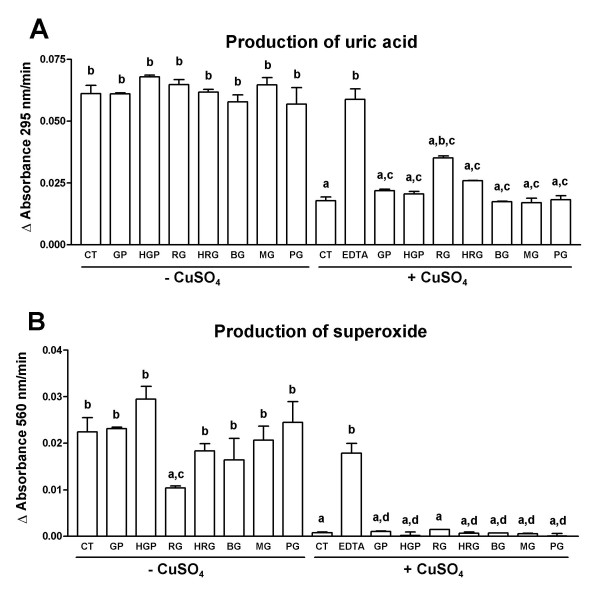
The Cu^2+^-chelating properties of aqueous garlic extracts were assessed using an approach based upon restoring the activity of xanthine oxidase which has been inhibited in the presence of 0.050 mM CuSO_4_. The activity of xanthine oxidase can be assessed by monitoring either the production of superoxide anion (measuring the reduction of NBT at 560 nm) or the formation of uric acid (following absorbance at 295 nm). Xanthine oxidase activity would be restored if the garlic extracts were able to chelate Cu^2+^. Panel A shows xanthine oxidase activity measuring uric acid production at 295 nm. ^a^p < 0.0001 vs. CT (-Cu^2+^), ^b^p < 0.0001 vs. CT (+Cu^2+^), ^c^p < 0.0001 vs. its respective group without Cu^2+^. Panel B shows xanthine oxidase activity measuring superoxide production by NBT reduction at 560 nm. ^a^p < 0.0001 vs. CT (-Cu^2+^); ^b^p < 0.0001 and ^c^p < 0.05 vs. CT (+Cu^2+^); ^d^p < 0.05 vs. its respective group without Cu^2+^. CT = Control (-Cu^2+^, +Cu^2+^), GP = aqueous extracts of garlic powder, HGP = heated aqueous extracts of garlic powder, RG = aqueous extracts of raw garlic, HRG = heated aqueous extracts of raw garlic, BG = aqueous extracts of boiled garlic, MG = aqueous extracts of microwave-treated garlic, and PG = aqueous extracts of pickled garlic. GP and HGP = 0.1 mg/mL, RG, HRG, BG, MG, and PG = 0.5 mg/mL. Data are mean ± SEM of 3 determinations, except for both CT groups and EDTA group in which n = 7.

To investigate if the ability of garlic extracts to inhibit Cu^2+^-induced lipoprotein oxidation in human serum was secondary to Cu^2+^-chelation, these extracts were tested in an *in vitro *system (see material and methods) to know if they were able to chelate Cu^2+^. The results for each extract at the higher concentration are presented in Fig. 8. Panel A shows uric acid production measured at 295 nm and panel B shows superoxide production measured by the NBT reduction at 560 nm. Cu^2+^-induced a decrease in xanthine oxidase activity measured both by uric acid production at 295 nm which decreased 71% and by NBT reduction at 560 nm which decreased 96% (Fig. 8). Uric acid production and NBT reduction were restored by EDTA. The extracts were added at the following concentrations: GP and HGP = 0.1 mg/mL, and RG, HRG, BG, MG, and PG = 0.5 mg/mL. We have previously shown that at these concentrations, the extracts increased lag time and decreased AUC indicating that they were able to inhibit Cu^2+^-induced lipoprotein oxidation (Figs. 3,4,5,6). In absence of Cu^2+ ^(-CuSO_4_) the extracts were unable to modify uric acid production indicating that they were unable to modify xanthine oxidase activity. In absence of Cu^2+^, NBT reduction was decreased significantly by RG indicating that this extract quenched superoxide anion. In presence of Cu^2+ ^(+CuSO_4_) the extracts were unable to restore (a) uric acid production (p > 0.05 vs. CT without Cu^2+^), with the exception of RG which was able to restore it partially (p < 0.05 vs. CT without Cu^2+^), and (b) NBT reduction (p > 0.05 vs. CT without Cu^2+^). In summary, the extracts were unable to restore xanthine oxidase activity indicating that they do not chelate Cu^2+^. Only RG showed a weak Cu^2+^-chelating activity.

## Discussion

Garlic has been used for millennia in folk medicine of many cultures to treat cardiovascular diseases and other disorders [1-8]. It has been shown in many cases that the protective effect of garlic is associated with its antioxidant properties [7,8]. The antioxidant properties of some garlic extracts used in this work have been studied. It has been found that aqueous extract of raw garlic scavenges hydroxyl radicals [20,31,32] and superoxide anion [32], inhibits lipid peroxidation [20], LDL oxidation [9], the formation of lipid hydroperoxides [20,31,32], and *in vivo *enhances endogenous antioxidant system [33] and prevents oxidative stress to the heart [34,35]. Chronic administration of raw garlic homogenate increases catalase and superoxide dismutase in rat heart [33] and protects heart against oxidative damage induced by adriamycin [34] or ischemia and reperfusion [35]. Aqueous extract of garlic powder are also able to scavenge hydroxyl radicals [36] and superoxide anion [37]. The heated aqueous extract of garlic powder maintains its ability to scavenge hydroxyl radicals [20]. In addition the ability of the aqueous extracts from boiled garlic cloves to scavenge hydroxyl radicals, superoxide anion, and hydrogen peroxide is not altered (unpublished results from our group). To our knowledge, additional antioxidant properties of the extracts from microwave-treated garlic cloves or from pickled garlic have not been studied.

Furthermore, the antioxidant properties of some isolated garlic compounds also have been studied. Allicin, the main component in aqueous extract from raw garlic and garlic powder, scavenges hydroxyl radicals and inhibit lipid peroxidation [38] and prevents the lung damage induced by ischemia-reperfusion [39]. The antioxidant properties of allicin may explain, at least in part, the ability of these extracts (from raw garlic or garlic powder) to inhibit Cu^2+^-induced lipoprotein oxidation in human serum. Alliin, the main component in extracts from boiled garlic cloves, microwave-treated garlic cloves and pickled garlic, scavenges hydroxyl radicals [40], hydrogen peroxide [41], and inhibits lipid peroxidation [41] and LDL oxidation [42]. This may explain, at least in part, the ability of boiled garlic, microwave-treated garlic, or picked garlic to inhibit Cu^2+^-induced lipoprotein oxidation in human serum. Interestingly, it has been shown that another garlic compounds such as S-allylcysteine [10,13], N-acetyl-S-allylcysteine [10], S-allylmercaptocysteine [10], alliin [10], allixin [10], and S-ethylcysteine, N-acetylcysteine, diallyl sulfide, and diallyl disulfide [14] are able to inhibit Cu^2+^-induced LDL oxidation. The antioxidant properties of S-allylcysteine [43], S-allylmercaptocysteine [44], diallyl sulfide [45], and diallyl disulfide [46] also have been seen *in vivo *in an experimental model of nephrotoxicity induced by gentamicin.

Our data strongly suggest that the ability of garlic to prevent Cu^2+^-induced lipoprotein oxidation in human serum is preserved in spite of inactivation of allinase by boiling, microwave or pickling or by the heating of garlic extracts and that the compound(s) involved in the inhibition of Cu^2+^-induced lipoprotein oxidation are heat stable. Our data are in contrast with previous studies in the literature showing that the heating may impair significantly several garlic properties. For example, microwave-treatment for 1 min impaired the anticancer properties of garlic [17] and the heating of garlic cloves by 15 min impairs significantly its ability to inhibit thromboxane B_2 _synthesis [19], and platelet aggregation [22], and the cyclooxygenase activity [18]. The heating by 10 min at 100°C reduced the bactericidal activity against *Helicobacter pylori *[21]. Interestingly, Kasuga *et al*. [47] have found that garlic extracts, prepared from boiled cloves, show efficacy in the following three experimental models: testicular hypogonadism induced by warm water treatment, intoxication of acetaldehyde, and growth of inoculated tumor cells, and Prasad *et al*. [20] found that the heating did not modify the ability of garlic extract to scavenge hydroxyl radicals. The data from Prasad *et al*. [20] and Kasuga *et al*. [47] strongly suggest that some garlic properties may remain unmodified after heating. Our data are in agreement with those of Prasad *et al*. [20] suggesting that the ability to inhibit Cu^2+^-induced lipoprotein oxidation is also preserved after the heating of garlic.

In addition, it was found that the garlic extracts used in our study were unable to chelate Cu^2+ ^suggesting that the ability of these extracts to inhibit Cu^2+^-induced lipoprotein oxidation is not secondary to Cu^2+^-chelation. Only RG showed a weak Cu^2+^-chelation activity, which was more evident at 295 nm. Based on previous data with aged garlic extracts [11] and some individual garlic compounds such as S-ethylcysteine, N-acetylcysteine, diallyl sulfide, and diallyl disulfide [14], we expected that our garlic extracts had Cu^2+^-chelating activity. The discrepancy with our data may simply reflect differences in composition in each garlic extract. This is additionally supported by the fact that the diethyl ether extract from aged garlic extract has no Cu^2+^-chelating activity [11]. The precise mechanism by which our extracts inhibit Cu^2+^-induced lipoprotein oxidation remains to be studied.

## Conclusions

(a) the heating of aqueous extracts of raw garlic or garlic powder or the heating of garlic cloves by boiling, microwave or pickling do not affect garlic's ability to inhibit Cu^2+^-induced lipoprotein oxidation in human serum, and (b) this ability is not secondary to Cu^2+^-chelation.

## Authors's contributions

JPCH conceived, designed and coordinated the study and drafted the manuscript. MGO performed the studies on Cu^2+^-chelation and studied the effect of boiled garlic, picked garlic and garlic submitted to microwave heating on Cu^2+^-induced lipoprotein oxidation in human serum. GA developed in our laboratory the method to oxidize lipoproteins by Cu^2+ ^and performed studies with garlic extracts of raw garlic and garlic powder. LBE participated in the studies on Cu^2+^-chelation. MM participated in the blood obtention and clinical chemistry analyses of blood serum. ONMC participated in the statistical analyses and developed the method to analyze the Cu^2+ ^chelating properties of garlic extracts. All authors read and approved the final manuscript.

## Competing interests

None declared.
